# Development and validation of a predictive model for chronic or persistent immune thrombocytopenia in children incorporating anti-glycoprotein IIb antibody: a retrospective cohort study utilizing LASSO regression and bootstrap stability analysis

**DOI:** 10.3389/fped.2026.1832712

**Published:** 2026-06-05

**Authors:** Ximei Yang, Xianghui Wan, Lei Ying, Xiaoying Chen, Zhiqiang Liu, Wei Huang, Jiangwei Ke

**Affiliations:** 1 Department of Laboratory Medicine, Jiangxi Provincial Children's Hospital, Nanchang, China; 2 Department of Laboratory Medicine, Jiangxi Provincial Cancer Hospital, Nanchang, China

**Keywords:** bootstrap stability analysis, children, immune thrombocytopenia, LASSO regression, nomogram, platelet glycoprotein antibody, predictive model, prognosis

## Abstract

**Background:**

Pediatric immune thrombocytopenia (ITP) is an autoimmune condition involving platelet destruction. While most cases resolve spontaneously, 20%–30% progress to chronic disease (cITP), necessitating early risk prediction. Current prognostic models for childhood ITP lack platelet-specific immunological markers. This study aimed to develop and validate a predictive model for chronic/persistent ITP(c/pITP) integrating clinical factors and anti-platelet glycoprotein antibodies.

**Methods:**

We retrospectively analyzed 381 children with newly diagnosed ITP (2022–2024). Patients were randomly allocated into training (*n* = 266) and test (*n* = 115) sets (7:3 ratio, seed = 42). Missing data were addressed via multiple imputation (m = 5). Predictors were selected through univariate analysis (*P* < 0.1), LASSO regression with 10-fold cross-validation, and bootstrap stability assessment (λ.1se, B = 1,000, selection frequency ≥ 90%). Candidate models were compared using bootstrap optimism correction and the Equivalent Parsimony Principle. The final model was evaluated on the independent test set for discrimination (AUC), calibration (Hosmer–Lemeshow test), and clinical utility (decision curve analysis).

**Results:**

Fourteen highly stable predictors were identified; the top three were platelet count (PLT, 100%), recent infection or vaccination history (IOV, 100%), and anti-GPIIb antibody status (99.9%). The final 4-variable model (age, IOV, PLT, anti-GPIIb) demonstrated good discrimination in the test set (AUC = 0.743; 95% CI: 0.650–0.836), with no evidence of overfitting (training AUC = 0.732). Calibration was satisfactory (Hosmer–Lemeshow *P* = 0.859). At a low-risk threshold (predicted probability ≤ 15%), the negative predictive value reached 94.7%, facilitating the rule-out of chronic progression. Decision curve analysis confirmed positive net benefit across threshold probabilities of 0%–60%.

**Conclusion:**

This concise model incorporating anti-GPIIb antibody enables early risk stratification, primarily to identify children unlikely to develop c/pITP.

## 1 Introduction

Immune thrombocytopenia (ITP) is an autoimmune disorder characterized by the immune-mediated destruction of platelets and compromised platelet production (1–3). While most pediatric cases are transient, approximately 20%–30% progress to chronic ITP (cITP), defined as persistence beyond 12 months (4). Chronic disease leads to sustained thrombocytopenia, increased bleeding risk, and treatment-related complications, such as those from prolonged corticosteroid use (5, 6). Early identification of children at risk for chronic progression is crucial for personalized management and to mitigate treatment-associated morbidity. Current prognostic tools for childhood ITP are limited. Advances in predictive analytics present opportunities for precision medicine (7, 8); however, existing models have not incorporated platelet-specific immunological markers. We hypothesized that integrating clinical characteristics, laboratory parameters, and anti-platelet glycoprotein antibodies would enhance the prediction of chronic or persistent ITP (c/pITP). This study aimed to develop and validate such a predictive model using rigorous statistical methods, including LASSO regression and bootstrap stability analysis.

## 2 Materials and methods

### 2.1 Study design and population

This retrospective cohort study analyzed children newly diagnosed with ITP through the hospital scientific research platform database. Using ICD-10 codes D69.3 and D69.4, we screened 1,144 records from January 2022 to December 2024. After excluding 281 duplicates, 863 unique patients were evaluated; of these, 482 were excluded due to incomplete history (*n* = 247), follow-up <12 months (*n* = 227), or alternative diagnoses (*n* = 8), leaving 381 patients. We acknowledge that ICD-10 coding, particularly D69.4 (other primary thrombocytopenia), carries inherent heterogeneity and may include non-ITP etiologies (e.g., inherited thrombocytopenias). To mitigate this, all screened records underwent manual chart review to confirm ITP diagnosis per international consensus criteria (9) prior to inclusion. The patient selection flowchart and baseline characteristics are presented in Supplementary Figure 1 and Supplementary Table 1, respectively. ITP diagnosis followed international consensus criteria (9). The ethics committee approved the study (JXSETYY-YXKY-20230005) and waived informed consent.

### 2.2 Data collection and variable definitions

Baseline demographic, clinical, and laboratory data were extracted from electronic medical records. Candidate predictors included age, sex, absolute lymphocyte count (ALC, ×10^9^/L), platelet-to-lymphocyte ratio (PLR), Helicobacter pylori antibody (HPAb) status, immunoglobulin levels (IgG, IgA, IgM, IgE), complement levels (C3, C4), platelet-associated immunoglobulin (PAIg), coagulation parameters [prothrombin time (PT), activated partial thromboplastin time (APTT), thrombin time (TT), international normalized ratio (INR), and fibrinogen (FIB)], antinuclear antibodies (ANA/ANAs), and anti-platelet glycoprotein antibodies (GPIb, GPIIb, GPIIIa, GPIX, GMP140) measured by flow cytometry.

Anti-platelet glycoprotein antibodies were detected by flow cytometric immunobead assay (Anti-Platelet Antibody Detection Kit, Suzhou YuanDe WeiKang Biotechnology Co., Ltd., China). EDTA-anticoagulated plasma was collected at diagnosis. Platelet-rich plasma was isolated, incubated with glycoprotein-coated microbeads, and labeled with FITC-conjugated anti-human IgG. Samples were acquired on a BD FACSCalibur flow cytometer. Positivity was defined as MFI ratio >1.9 for GPIb and >1.5 for other glycoproteins. IOV was defined as infection or vaccination within 4 weeks preceding diagnosis.

### 2.3 Outcome definition

The primary outcome was disease status at 12 months: nITP (platelet normalization within 3 months without relapse), persistent ITP (3–12 months), or chronic ITP (>12 months) (10). Persistent and chronic ITP cases were grouped into a single category, designated as c/pITP (y = 1), and compared against nITP (y = 0).

### 2.4 Missing data handling

Variables with >30% missing data were excluded. The remaining data were split into training (70%) and test (30%) sets (11) using stratified random sampling (seed = 42). Missing values in the training set were imputed by multiple imputation (MICE, m = 5, maxit = 50). LASSO regression, bootstrap stability assessment, and candidate model comparison were performed independently on each imputed dataset; final coefficients, selection frequencies, and optimism-corrected metrics were pooled using Rubin's rules. Test set missing values were imputed using predictive means derived from the training imputation model to ensure independent validation. Sensitivity analyses confirmed robustness across imputation strategies (Supplementary Table 2).

### 2.5 Statistical analysis and model building

Descriptive statistics were calculated. Continuous variables were compared using the Mann–Whitney *U* test and categorical variables using *χ*^2^ or Fisher's exact test. A two-sided *P* < 0.05 was considered significant.

A multi-phase analytical framework was implemented exclusively in the training set to prevent data leakage. Variable selection involved three steps: (i) univariate logistic regression (*P* < 0.10); (ii) LASSO regression with 10-fold cross-validation; and (iii) bootstrap stability assessment (B = 1,000, λ.1se criterion, selection frequency ≥90%; see Supplementary Methods 1 for detailed resampling protocol). Candidate models incorporating clinically relevant variable combinations were compared using bootstrap optimism correction and the Equivalent Parsimony Principle (ΔCorrected AUC < 0.01). The final model was evaluated on the independent test set (Phase 4).

### 2.6 Model validation

Discrimination was quantified by AUC. Calibration was assessed by Hosmer–Lemeshow goodness-of-fit test and calibration plots (12). Clinical utility was evaluated by decision curve analysis (DCA) (13). Diagnostic performance (sensitivity, specificity, PPV, NPV, accuracy, LR+/LR−) was calculated at the optimal threshold (0.30) and additional thresholds (0.15–0.60).

### 2.7 Nomogram development

Regression coefficients from the final model were transformed to a 0–100 point scale to construct a clinical nomogram.

### 2.8 Software

Analyses were conducted using R 4.3.0 (packages: pROC v1.18.0, rms v6.7.0, rmda v1.6) and Python 3.9 (libraries: scikit-learn v1.3.0, statsmodels v0.14.0).

## 3 Results

### 3.1 Patient characteristics

Of the 381 pediatric patients included (Table 1), 255 (66.9%) had newly diagnosed immune thrombocytopenia (nITP), while 126 (33.1%) had chronic/persistent ITP (c/pITP). The median age was 36.00 months (interquartile range, IQR: 10.67–72.00), and 230 (60.4%) were male. Significant differences were observed between the groups: c/pITP patients were older (median 60.00 vs. 24.00 months, *P* < 0.001), had a higher proportion of females (49.21% vs. 34.90%, *P* = 0.007), presented with higher platelet counts (14.50 vs. 9.00 × 10^9^/L, *P* = 0.003), exhibited a lower frequency of infection onset within one month (IOV) (14.29% vs. 25.10%, *P* = 0.016), and demonstrated lower positivity for anti-glycoprotein IIb (anti-GPIIb) antibodies (34.92% vs. 47.45%, *P* = 0.020). The training (*n* = 266) and test (*n* = 115) sets were well-balanced for the outcome distribution (c/pITP prevalence: 33.1% vs. 33.0%, *P* = 0.983).

**Table 1:** Baseline characteristics of ITP patients stratified by disease course.

Variables	Total (*n* = 381)	nITP (*n* = 255)	c/pITP (*n* = 126)	Z/U value	*P* value
Age, month	36.00 (10.67, 72.00)	24.00 (5.07, 60.00)	60.00 (24.00, 96.00)	−6.91	**<0**.**001**
Sex, *n* (%)				7.21	**0**.**007**
male	230 (60.37)	166 (65.10)	64 (50.79)		
female	151 (39.63)	89 (34.90)	62 (49.21)		
IOV, *n* (%)				5.84	**0**.**016**
Yes	82 (21.52)	64 (25.10)	18 (14.29)		
No	299 (78.48)	191 (74.90)	108 (85.71)		
HPAb, *n* (%)				0.29	0.591
Positive	47 (13.86)	32 (14.61)	15 (12.50)		
Negative	292 (86.14)	187 (85.39)	105 (87.50)		
ANA, *n* (%)				0.88	0.348
Positive	50 (13.77)	30 (12.55)	20 (16.13)		
Negative	313 (86.23)	209 (87.45)	104 (83.87)		
ANAs, *n* (%)				1.73	0.189
Positive	16 (4.38)	13 (5.39)	3 (2.42)		
Negative	349 (95.62)	228 (94.61)	121 (97.58)		
PAIg, *n* (%)				0.60	0.439
Positive	158 (65.02)	97 (62.99)	61 (68.54)		
Negative	85 (34.98)	57 (37.01)	28 (31.46)		
GPIb, *n* (%)				2.55	0.110
Positive	85 (22.31)	63 (24.71)	22 (17.46)		
Negative	296 (77.69)	192 (75.29)	104 (82.54)		
GPIIb, *n* (%)				5.39	**0**.**020**
Positive	165 (43.31)	121 (47.45)	44 (34.92)		
Negative	216 (56.69)	134 (52.55)	82 (65.08)		
GPIIIa, *n* (%)				0.78	0.377
Positive	38 (9.97)	23 (9.02)	15 (11.90)		
Negative	343 (90.03)	232 (90.98)	111 (88.10)		
GMP140, *n* (%)				2.28	0.131
Positive	169 (44.36)	120 (47.06)	49 (38.89)		
Negative	212 (55.64)	135 (52.94)	77 (61.11)		
GPIX, *n* (%)				0.45	0.504
Positive	77 (20.21)	54 (21.18)	23 (18.25)		
Negative	304 (79.79)	201 (78.82)	103 (81.75)		
PLT, ×10^9^/L	11.00 (3.00, 25.00)	9.00 (3.00, 23.00)	14.50 (6.00, 28.25)	−2.96	**0**.**003**
ALC, ×10^9^/L	3.78 (2.56, 5.92)	4.33 (2.76, 6.38)	3.07 (2.21, 4.54)	4.66	**<0**.**001**
PLR	2.78 (0.86, 7.64)	2.07 (0.66, 6.71)	4.73 (1.67, 9.25)	−4.50	**<0**.**001**
IgA, g/L	0.80 (0.36, 1.32)	0.68 (0.28, 1.20)	1.04 (0.69, 1.42)	−4.64	**<0**.**001**
IgG, g/L	7.62 (5.82, 9.76)	7.23 (5.18, 9.56)	8.42 (6.67, 9.92)	−3.70	**<0**.**001**
IgM, g/L	1.04 (0.72, 1.47)	0.98 (0.67, 1.43)	1.13 (0.90, 1.59)	−3.36	**<0**.**001**
IgE, IU/mL	83.96 (35.95, 239.06)	87.19 (36.05, 249.10)	79.00 (34.05, 198.00)	−0.29	0.775
C3, g/L	1.01 (0.90, 1.15)	1.00 (0.89, 1.15)	1.04 (0.94, 1.13)	−1.03	0.304
C4, g/L	0.21 (0.17, 0.27)	0.21 (0.17, 0.26)	0.22 (0.18, 0.28)	−1.22	0.223
APTT, s	32.60 (29.30, 36.30)	32.60 (29.05, 36.55)	32.50 (29.60, 35.30)	−0.51	0.609
PT, s	12.00 (11.40, 12.60)	11.90 (11.40, 12.60)	12.20 (11.65, 12.70)	−2.69	**0**.**007**
TT, s	16.50 (15.30, 17.80)	16.90 (15.65, 18.10)	16.00 (14.90, 16.90)	−4.36	**<0**.**001**
FIB, g/L	2.37 (1.93, 2.89)	2.28 (1.86, 2.82)	2.50 (2.03, 2.99)	−2.74	**0**.**006**
INR	1.07 (1.02, 1.14)	1.06 (1.01, 1.14)	1.10 (1.04, 1.16)	−3.05	**0**.**002**

Data are presented as median (interquartile range) for continuous variables and *n* (%) for categorical variables. ITP, immune thrombocytopenia; nITP, newly diagnosed ITP; c/pITP, chronic or persistent ITP; IOV, history of infection or vaccination within 4 weeks preceding ITP diagnosis; HPAb, *Helicobacter pylori* antibody; ANA, antinuclear antibody; ANAs, antinuclear antibodies (nRNP, Sm, SS-A60, SS-Ro52, SS-B, Scl-70, PM-Scl, Jo-1, CENP B, PCNA, dsDNA, Histones, nucleosome, ribosomal P protein, AMA M2); PAIg, platelet-associated immunoglobulin; GPIb/IIb/IIIa/IX, glycoprotein Ib/IIb/IIIa/IX; GMP140, P-selectin; PLT, platelet count; ALC, absolute lymphocyte count; PLR, platelet-to-lymphocyte ratio; IgA/IgG/IgM/IgE, immunoglobulin A/G/M/E; C3/C4, complement component 3/4; APTT, activated partial thromboplastin time; PT, prothrombin time; TT, thrombin time; FIB, fibrinogen; INR, international normalized ratio. PAIg data available in 243 patients (138 missing, 36.2%).

Bold values indicate statistically significant differences (*P* < 0.05) between the nITP and c/pITP groups.

### 3.2 Missing data

The percentages of missing data were as follows: platelet-associated immunoglobulin (PAIg) 36.2% (excluded from analysis), Helicobacter pylori antibody (HPAb) 11.0%, antinuclear antibody (ANA) 4.7%, antinuclear antibodies (ANAs) 4.2%, immunoglobulins and complement components 2.1%, and coagulation tests 1.1%. Sensitivity analysis of four imputation methods (MICE_Bayesian, MICE_Point, Simple_Median, Simple_Mean) showed coefficient of variation <5% for core prognostic factors, confirming robustness. HPAb stability varied by method (CV 18.5%), leading to its exclusion from the final model (Supplementary Table 2). The test set AUC demonstrated robustness, varying by less than 0.015 (range: 0.703–0.715) across imputation approaches.

### 3.3 Variable selection

**Figure 1:**
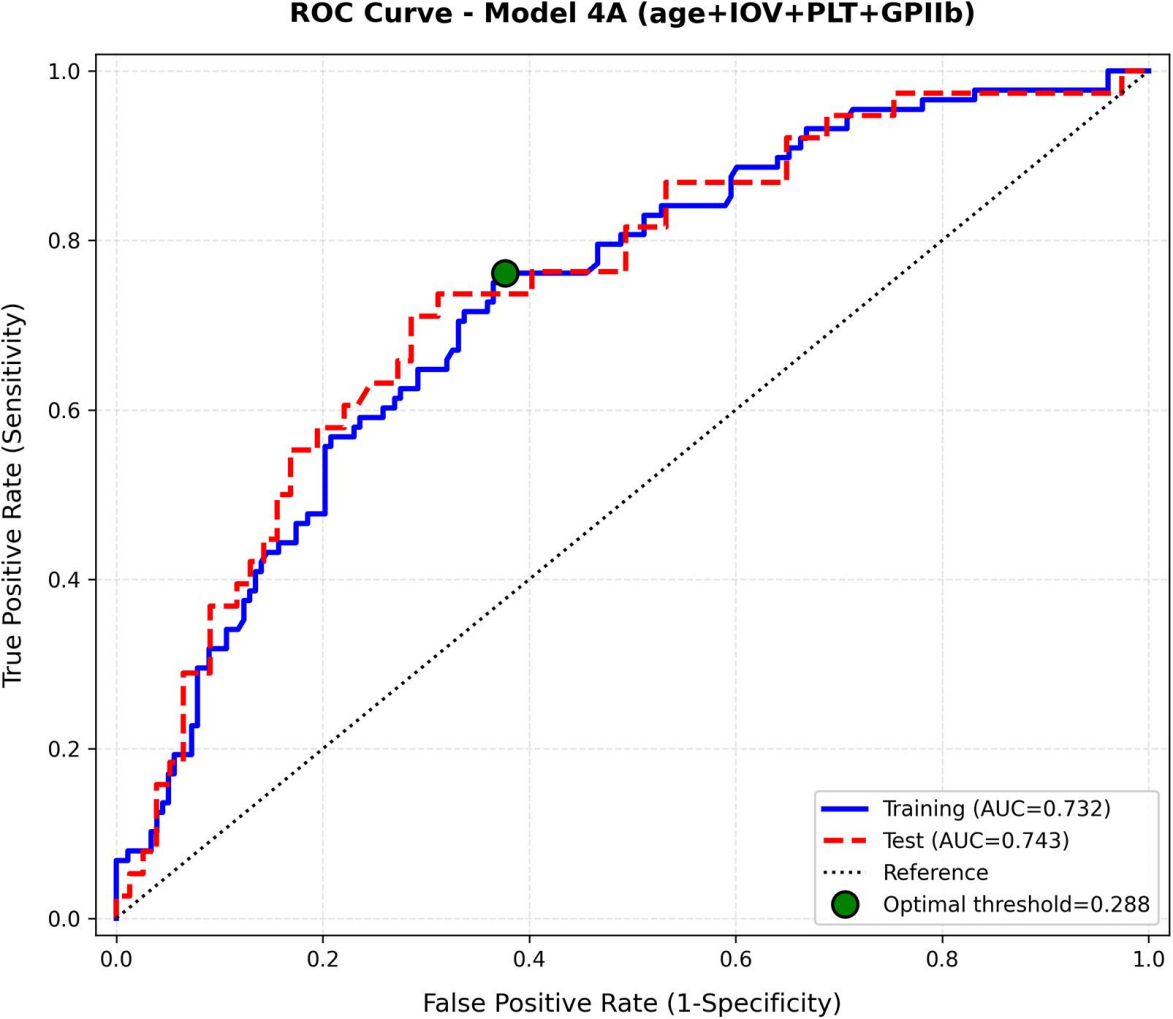
ROC curves for the final 4-variable prediction model in the training set (AUC 0.732, 95% CI 0.670–0.792) and test set (AUC 0.743, 95% CI 0.650–0.836). The minimal difference (ΔAUC = −0.011) indicates good generalization without overfitting.

Fourteen variables exhibited *P* values <0.1 in univariate analysis (Table 2). LASSO regression (λ.1se) identified fourteen variables with non-zero coefficients. Subsequent bootstrap stability analysis identified fourteen highly stable variables (selection frequency ≥90%), indicating exceptional robustness (Table 3). The most stable predictors included PLT (100%), IOV (100%) and anti-GPIIb antibody status (99.9%). The best-performing model (TOP 8) was selected based on these stable variables. Complete comparison metrics for all candidate models are provided in Supplementary Table 3. These stable variables and statistical performance metrics were compared with six models incorporating clinically relevant presets in total (Table 4). The M-4A (PLT + IOV + GPIIb + age) demonstrated an optimism-corrected AUC of 0.702 (95% CI: 0.567–0.838), which was within 0.01 of the highest-performing model (M-6, corrected AUC = 0.711). Adhering to the principle of parsimony and prioritizing clinical feasibility (given that GPIIb is a disease-specific marker, whereas TT and C4 are less specific), M-4A was selected as the final model. Figure 1 presents the ROC curves for the final 4-variable model (M-4A) in both the training and test sets. The model achieved an AUC of 0.732 (95% CI: 0.670–0.792) in the training set and 0.743 (95% CI: 0.650–0.836) in the independent test set. The minimal difference (ΔAUC = −0.011, training-test) confirms the absence of overfitting and supports excellent generalizability to unseen pediatric populations.

**Table 2:** Univariable logistic regression analysis in the training set.

Variable	OR (95%CI)	*P*	Included in multivariable analysis
**Age, months**	**1.015** (**1.008**–**1.021)**	**<0**.**001**	**Yes**
**ALC, ×10** ^9^ **/L**	**0.807** (**0.715**–**0.911)**	**<0**.**001**	**Yes**
**TT, s**	**0.773** (**0.665**–**0.898)**	**<0**.**001**	**Yes**
**IgA, g/L**	**1.882** (**1.259**–**2.814)**	**0**.**002**	**Yes**
**GPIIb (positive vs. negative)**	**0.449** (**0.264**–**0.766)**	**0**.**003**	**Yes**
**C4, g/L**	**50.276** (**2.400−1,053.273)**	**0**.**012**	**Yes**
**GMP140 (positive vs. negative)**	**0.522** (**0305**–**0.893)**	**0**.**018**	**Yes**
**Sex (male vs. female)**	**0.546** (**0.324**–**0.918)**	**0**.**022**	**Yes**
**FIB, g/L**	**1.532** (**1.056**–**2.221)**	**0**.**025**	**Yes**
**IOV (Yes vs. no)**	**0.453** (**0.226**–**0.908)**	**0**.**026**	**Yes**
**PT, s**	**1.260** (**1.003**–**1.584)**	**0**.**047**	**Yes**
**PLR**	**1.028** (**1.000**–**1.057)**	**0**.**049**	**Yes**
**INR**	**14.127** (**1.006−198.346)**	**0**.**049**	**Yes**
**PLT, ×10** ^9^ **/L**	**1.012** (**0.999**–**1.024)**	**0**.**063**	**Yes**
IgG, g/L	1.055 (0.984–1.130)	0.130	No
C3, g/L	2.630 (0.726–9.531)	0.141	No
GPIb (positive vs. negative)	0.639 (0.320–1.272)	0.202	No
APTT, s	0.969 (0.920–1.021)	0.237	No
HPAb	0.615 (0.265–1.432)	0.260	No
ANA (positive vs. negative)	1.492 (0.736–3.023)	0.267	No
GPIX (positive vs. negative)	0.757 (0.391–1.467)	0.409	No
ANAs (positive vs. negative)	0.645 (0.169–2.469)	0.522	No
GPIIIa (positive vs. negative)	0.741 (0.279–1.964)	0.547	No
IgE, IU/mL	1.000 (0.999–1.001)	0.776	No
IgM, g/L	0.992 (0.937–1.050)	0.778	No

OR, odds ratio; CI, confidence interval; ALC, absolute lymphocyte count; TT, thrombin time; IgA/IgG/IgM/IgE, immunoglobulin A/G/M/E; GPIb/IIb/IIIa/IX, glycoprotein Ib/IIb/IIIa/IX; GMP140, P-selectin; FIB, fibrinogen; IOV, history of infection or vaccination within 4 weeks preceding ITP diagnosis; PLR, platelet-to-lymphocyte ratio; INR, international normalized ratio; PT, prothrombin time; PLT, platelet count; C3/C4, complement component 3/4; HPAb, *Helicobacter pylori* antibody; APTT, activated partial thromboplastin time; ANAs, antinuclear antibodies (nRNP/Sm, Sm, SS-A60, SS-Ro52, SS-B, Scl-70, PM-Scl, Jo-1, CENP B, PCNA, dsDNA, Histones, nucleosome, ribosomal P protein, AMA M2). Variables with *P* < 0.10 were selected for LASSO regression and subsequent multivariable model building. Analysis performed in training set (*n* = 266).

Bold “Yes” indicates variables selected for subsequent LASSO regression and multivariable model building (*P* < 0.10).

**Table 3:** Stability analysis of bootstrap (B = 1,000, λ.1se criterion).

Sorting	Variables	Selection frequency	Coefficient (Mean ± SD)	OR (95% CI)	Stability level
1	age	99.3%	0.375 ± 0.230	1.444 (0.962−2.402)	Highly stable
2	ALC	99.0%	−0.340 ± 0.247	0.719 (0.430−1.126)	Highly stable
3	TT	99.7%	−0.450 ± 0.215	0.639 (0.410−0.950)	Highly stable
4	IgA	99.5%	0.883 ± 1.203	1.339 (0.639–47.784)	Highly stable
5	GPIIb	99.9%	−0.316 ± 0.171	0.734 (0.517–0.994)	Highly stable
6	C4	99.8%	0.301 ± 0.195	1.335 (0.922–1.990)	Highly stable
7	GP140	99.3%	−0.249 ± 0.164	0.779 (0.560–1.058)	Highly stable
8	sex	99.6%	−0.264 ± 0.164	0.768 (0.561–1.063)	Highly stable
9	FIB	98.5%	−0.176 ± 0.218	0.844 (0.542–1.288)	Highly stable
10	IOV	100.0%	−0.406 ± 0.177	0.668 (0.469–0.930)	Highly stable
11	PT	98.5%	0.173 ± 0.427	1.241 (0.407–2.640)	Highly stable
12	PLR	99.2%	−0.351 ± 0.262	0.781 (0.388–1.108)	Highly stable
13	INR	97.2%	−0.096 ± 0.401	0.873 (0.455–2.569)	Highly stable
14	PLT	100.0%	0.646 ± 0.254	1.889 (1.215–3.331)	Highly stable

ALC, absolute lymphocyte count; TT, thrombin time; IgA, immunoglobulin A; GPIIb, glycoprotein IIb; C4, complement component 4; GMP140, P-selectin; FIB, fibrinogen; IOV, history of infection or vaccination within 4 weeks preceding ITP diagnosis; PT, prothrombin time; PLR, platelet-to-lymphocyte ratio; INR, international normalized ratio; PLT, platelet count.

**Table 4:** Bootstrap optimism correction of candidate prediction models.

Model	Variables	Apparent_AUC	Optimism	Corrected_AUC (95CI)	AIC	Corrected_Brier
TOP8	PLT + IOV + GPIIb + C4+TT + sex + IgA + age	0.771	0.069	0.702 (0.570–0.835)	302.04	0.208
M-4A	Age + IOV + PLT + GPIIb	0.732	0.029	0.702 (0.567–0.838)	308.46	0.205
M-4B	Age + IOV + PLT + sex	0.720	0.033	0.687 (0.551–0.823)	311.51	0.209
M-5A	Age + IOV + PLT + GPIIb + TT	0.746	0.036	0.711 (0.577–0.845)	304.05	0.202
M-5B	Age + IOV + PLT + GPIIb + sex	0.740	0.039	0.701 (0.567–0.834)	306.65	0.205
M-6	Age + IOV + PLT + GPIIb + TT + sex	0.754	0.043	0.711 (0.579–0.844)	302.63	0.202
M-8	Age + IOV + PLT + GPIIb + TT + sex + C4 + GMP140	0.761	0.051	0.710 (0.580–0.841)	301.81	0.204

AUC, area under the receiver operating characteristic curve; CI, confidence interval; AIC: Akaike Information Criterion (smaller is better, penalizing model complexity); PLT, platelet count; IOV, history of infection or vaccination within 4 weeks preceding ITP diagnosis; GPIIb, glycoprotein IIb; C4, complement component 4; TT, thrombin time; IgA, immunoglobulin A; GMP140, P-selectin; PLR, platelet-to-lymphocyte ratio. M-4A selected as final model based on equivalent parsimony principle.

### 3.4 Final predictive model

**Figure 2:**
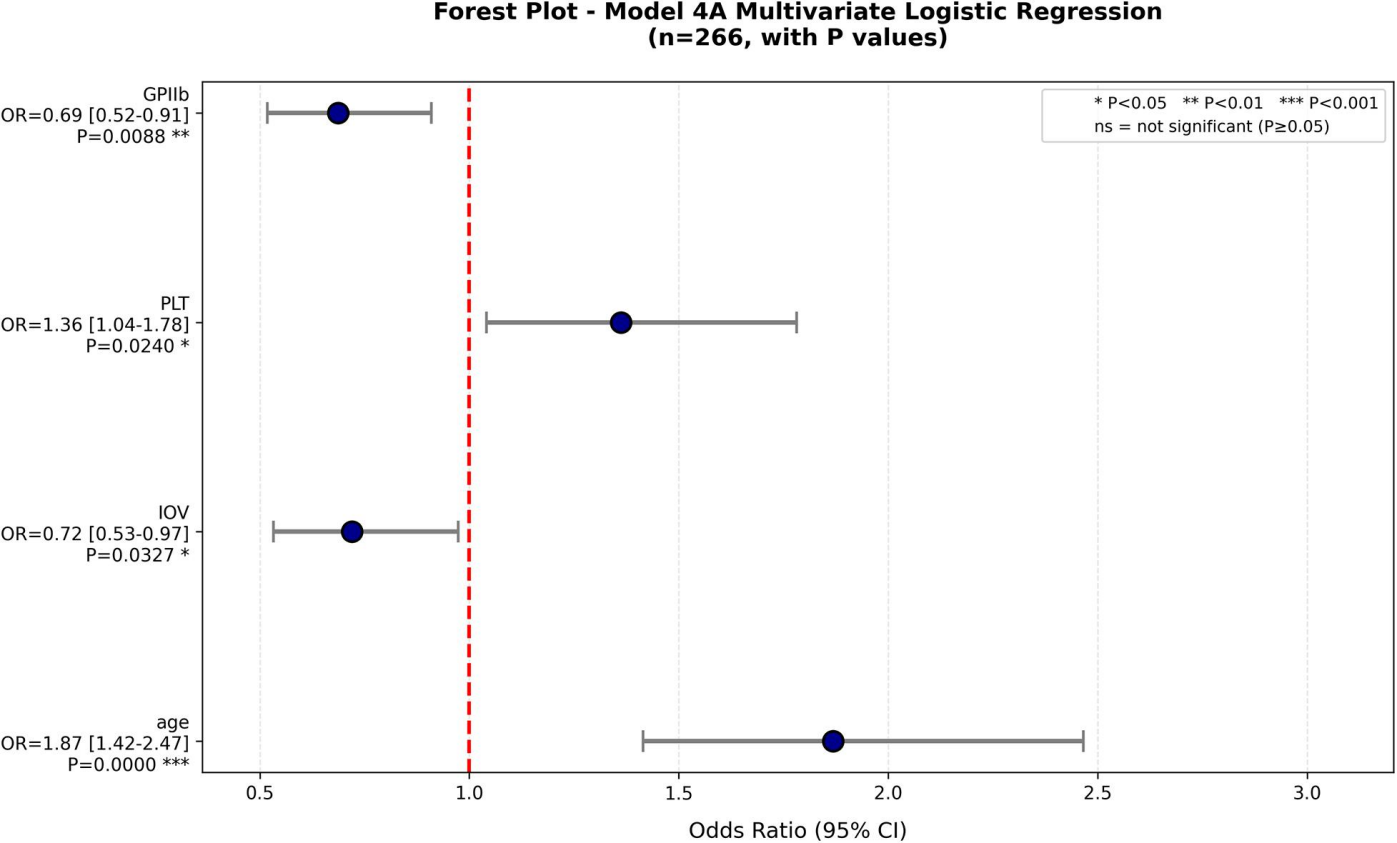
Forest plot of multivariate logistic regression analysis for predictors of chronic/persistent ITP. Adjusted odds ratios (ORs) and 95% confidence intervals (CIs) are shown for each predictor in the final 4-variable model (training set, *n* = 266). OR values and 95% CIs are rounded to two decimal places. *P < 0.05, **P < 0.01, ***P < 0.001 PLT, platelet count; IOV, recent infection or vaccination; GPIIb, anti-glycoprotein IIb antibody.

Multivariable analysis for the final model (training set) revealed that age was the strongest risk factor for chronic/persistent progression, with an odds ratio (OR) of 1.87 per month (95% CI: 1.42–2.47, *P* < 0.001) (Figure 2). A higher platelet count was associated with an increased risk of chronicity (OR 1.36, 95% CI: 1.04–1.78, *P* = 0.015). IOV was associated with reduced risk of chronic progression (OR 0.72, 95% CI: 0.53–0.97, *P* = 0.028). Positivity for anti-GPIIb antibodies was also protective (OR 0.69, 95% CI: 0.52–0.91, *P* = 0.010).

### 3.5 Model calibration and validation

**Figure 3:**
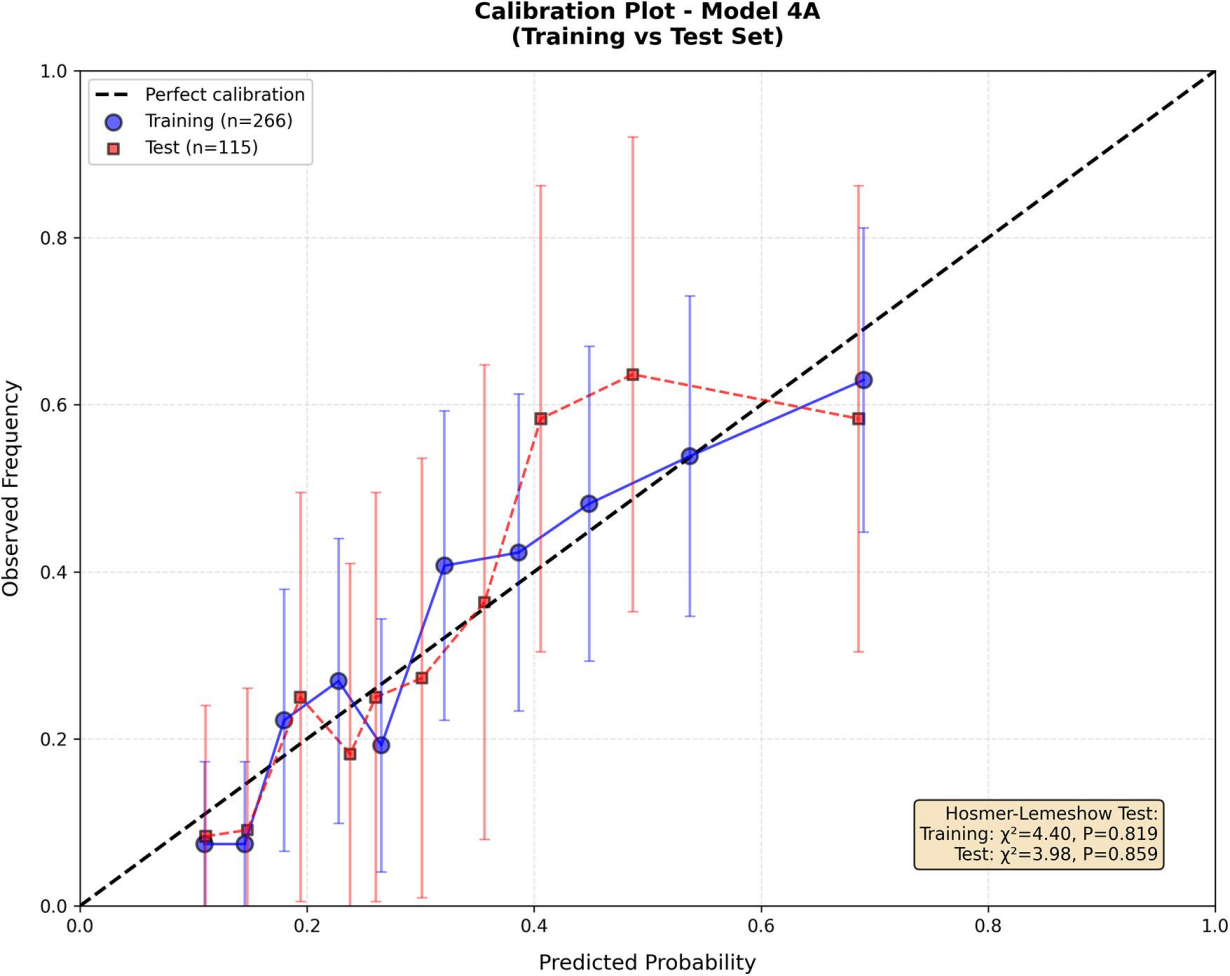
Calibration plots of the final prediction model. Calibration plots for the training set (blue) and test set (red). The dashed diagonal line represents perfect calibration. Solid lines with markers represent observed versus predicted probabilities across deciles. Calibration metrics: training set, slope 0.98, intercept −0.02, Brier score 0.187; test set, slope 0.97, intercept −0.01, Brier score 0.190. Hosmer–Lemeshow test *P* > 0.05 for both sets.

**Figure 4:**
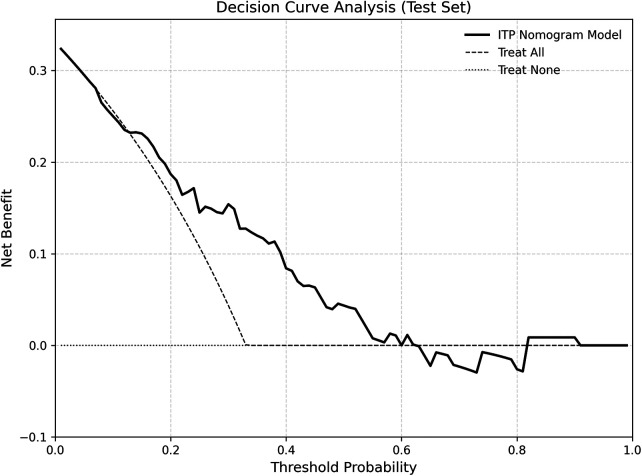
Decision curve analysis for the 4-variable model (M-4A) in the test set (*n* = 115). The *x*-axis indicates threshold probability (Pt); the *y*-axis shows net benefit. The model (solid line) yields positive net benefit across Pt = 0.00–0.60 (shaded area), outperforming “treat all” (dashed line) and “treat none” (horizontal line) strategies.

The model demonstrated good calibration, as evidenced by non-significant Hosmer–Lemeshow test results in both the training and test sets (training: *χ*^2^ = 4.40, *P* = 0.819; test: χ^2^ = 3.98, *P* = 0.859) (Figure 3). At the optimal threshold of 0.30, the model’s performance was: sensitivity 73.7%, specificity 68.8%, PPV 53.8%, NPV 84.1%. At a lower threshold of 0.15 (corresponding to a nomogram score of ∼20), the model achieved a sensitivity of 97.4% and an NPV of 94.7%, supporting exclusion of c/pITP (Table 5). Decision curve analysis (DCA) indicated a positive net benefit across a wide range of threshold probabilities (Pt = 0%–60%), outperforming both the “treat all” and “treat none” strategies (Figure 4). The highest net benefit was observed at Pt ≈ 0.25–0.30.

**Table 5:** Diagnostic performance of the final prediction model at various probability thresholds in the test set.

Threshold	Sensitivity	Specificity	PPV	NPV	Accuracy	Youden	LR+	LR−
**0**.**15**	**0.974**	**0.234**	0.385	**0**.**947**	**0.478**	**0.208**	1.271	0.113
0.2	0.895	0.351	0.405	0.871	0.530	0.245	1.378	0.300
0.25	0.763	0.520	0.439	0.816	0.600	0.283	1.588	0.456
**0**.**3**	**0**.**737**	**0**.**688**	**0**.**539**	**0**.**841**	**0**.**704**	**0**.**425**	**2**.**364**	**0**.**382**
0.35	0.632	0.753	0.558	0.806	0.713	0.385	2.560	0.489
0.4	0.447	0.857	0.607	0.759	0.722	0.305	3.132	0.645
0.5	0.290	0.922	0.647	0.725	0.713	0.212	3.715	0.771
0.6	0.158	0.948	0.600	0.695	0.687	0.106	3.039	0.888

Optimal threshold (0.30). PPV, positive predictive value; NPV, negative predictive value. LR+, positive likelihood ratio; LR−, negative likelihood ratio.

Bold values indicate the optimal threshold (0.30) determined by the Youden index, with the highest diagnostic accuracy.

### 3.6 Model application

**Figure 5:**
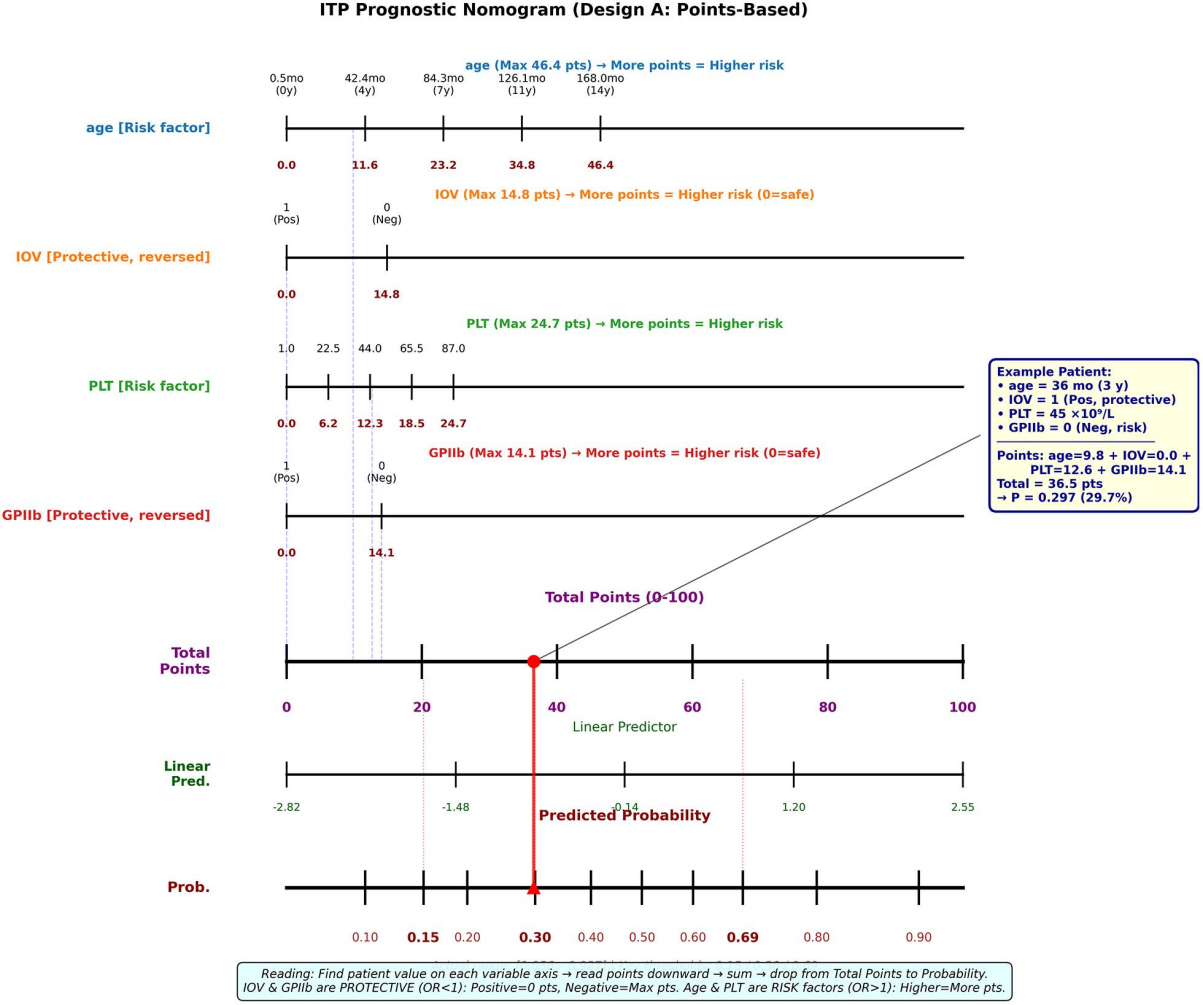
Nomogram for predicting chronic/persistent ITP. Nomogram for predicting chronic/persistent ITP. Points are assigned for each variable: age (0–46.4 points), platelet count (PLT) (0–24.7 points), IOV (0/14.8 points), and anti-GPIIb antibody status (0/14.1 points). Total scores range from 0 to 100, corresponding to predicted probabilities of approximately 10%–90%. Risk is categorized into two tiers: low risk (≤20 points, probability <15%) and elevated risk (>20 points, probability ≥15%), the latter indicating need for enhanced surveillance.

For clinical application, a nomogram was constructed using the logistic regression coefficients, assigning points as follows: Age (0–46.4 points), Platelet count (PLT) (0–24.7 points), IOV (0/14.8 points), and anti-GPIIb status (0/14.1 points), for a total possible score of 100 (Figure 5). Based on threshold analysis and clinical utility optimization, risk was categorized into two tiers: low risk (≤20 points, corresponding to a predicted probability <15%) and elevated risk (>20 points, probability ≥15%). The low-risk tier, capturing approximately 40%–50% of patients, is designed for safe rule-out of chronic progression (NPV 94.7% at the 0.15 threshold), while the elevated-risk tier indicates need for enhanced surveillance and structured follow-up.

## 4 Discussion

In this retrospective cohort study of 381 pediatric patients with newly diagnosed immune thrombocytopenia (ITP), we developed and validated a 4-variable predictive model that incorporates anti-GPIIb antibody status, selected from a panel of five platelet glycoprotein antibodies. The model demonstrated good discrimination (AUC 0.743), calibration, and clinical utility. We applied multiple imputation, bootstrap resampling with optimism correction, and an “equivalent parsimony” criterion for model selection to reduce overfitting. To our knowledge, this represents the first prognostic model for childhood ITP that incorporates anti-platelet glycoprotein antibodies together with these validation techniques.

### 4.1 Methodological innovation

We utilized a cytometric bead array to simultaneously detect five anti-platelet glycoprotein antibodies (GPIIb, GPIIIa, GPIb, GPIX, GMP140), based on the central pathophysiological role of humoral immune-mediated platelet destruction. While Chinese guidelines (14, 15) recommend anti-platelet glycoprotein antibody testing for diagnostic clarification, we acknowledge that ASH and international consensus guidelines (10, 16) currently advise against routine testing due to insufficient evidence for prognostic utility.

Our study does not advocate for universal adoption of antibody testing in all ITP patients. Rather, we demonstrate that when such testing is performed (e.g., in case of diagnostic uncertainty or research contexts), the anti-GPIIb result provides independent prognostic information. Before routine clinical implementation, cost-effectiveness analyses comparing antibody-assisted risk stratification against standard care are required, particularly in resource-limited settings. The model remains clinically useful even without antibody data (4B, AUC 0.687), offering flexibility based on local practice patterns.

The flow cytometric immunobead assay employed here demonstrated superior performance (73.13% sensitivity, 81.98% specificity) compared to the traditional monoclonal antibody immobilization of platelet antigens (MAIPA) assay, which showed a sensitivity of 41.46% (17). LASSO regression was applied to select the most informative predictor from the five candidate antibodies, effectively balancing the comprehensive antibody panel information with model parsimony. GPIIb (CD41) is the most abundant glycoprotein on the platelet membrane, constituting 15%–20% of its total membrane protein. As the α-subunit of the αIIbβ3 integrin, it represents the dominant autoantigen target in ITP, with 60%–80% of patients possessing anti-GPIIb/IIIa antibodies (18). Bootstrap stability analysis confirmed GPIIb as a highly stable predictor (selected in 99.9% of resamples), while GPIb and GPIIIa were consistently shrunk to zero, indicating weaker independent associations with chronic disease progression. The findings from Peking University People’s Hospital, which indicated that anti-GPIIb/IIIa positivity was associated with a reduced response to first-line therapy (42.1% vs. 72.1%, *P* < 0.05) (19), align with our observations. We initially constructed a base model using four highly stable variables (age, IOV, GPIIb, PLT; selection frequency ≥99%). The independent contribution of GPIIb was supported by its high stability and biological relevance; replacing it with sex (Model_4B) reduced the corrected AUC from 0.702 to 0.687, though the difference was not statistically significant (DeLong test *P* = 0.625). Nevertheless, we retained it to enhance pathophysiological interpretability.

### 4.2 Comparison with recent studies

Several predictive models for pediatric ITP have been reported recently. A study from Beijing Children's Hospital (2024) employed XGBoost with T-cell subsets, achieving an AUROC of 0.81–0.84 (20); however, this approach necessitates specialized flow cytometry analysis software. In contrast, our antibody-based methodology directly reflects ITP pathophysiology, utilizes fewer variables (4 vs. 12), and incorporates more rigorous Bootstrap validation. Research from Xinjiang Medical University (2025) utilized age, MPV, 25-(OH)D, and platelet recovery time, achieving an AUC of 0.865 (21), but required dynamic monitoring over a median of 14 days, thereby limiting its utility for early prediction. Our model relies exclusively on baseline data. A study from Soochow University (2024) integrated LASSO feature selection with a random forest classifier, reporting an AUC of 0.867 in a cohort of 156 patients (22), though it lacked external validation. Our study benefits from a larger sample size (n = 381) and robust Bootstrap internal validation (B = 1,000), enhancing methodological rigor. While our AUC (0.743) is lower than some reported values, it exceeds that reported by Schmidt et al. (0.70) (23). Predicting chronicity over 12 months inherently involves greater temporal uncertainty compared to short-term outcomes. Furthermore, our unselected cohort (platelet count 3–87 × 10^9^/L) demonstrated greater heterogeneity than the cohorts in studies restricted to specific subgroups. Although Bootstrap validation is rigorous, it may still yield optimistic performance estimates; thus, external validation remains essential.

### 4.3 Predictor interpretation

The protective effect of anti-GPIIb positivity (OR 0.69) requires careful interpretation. Antibodies against GPIIb/IIIa (the fibrinogen receptor) can be of any immunoglobulin class (IgG, IgM, or IgA). Transient ITP is often associated with IgM-class anti-platelet antibodies, which are produced early in immune responses and may indicate acute infection-triggered, self-limiting autoimmunity (24). In contrast, IgG-class antibodies against specific epitopes of GPIIb/IIIa may indicate “classical” B-cell mediated autoimmunity responsive to immunomodulatory therapies (25). Our assay detected total immunoglobulin binding which is primarily IgG in chronic ITP. The protective association observed here likely reflects the detection of early, transient IgM responses that cross-react with the assay, or non-pathogenic IgG binding in patients with acute, infection-triggered ITP who are destined for spontaneous resolution. Future studies should differentiate antibody classes (IgG vs. IgM) and glycoprotein specificity combinations (GPIIb + GPIIIa co-positivity) to refine risk stratification. Antibody detection at diagnosis may capture transient immune activation. Age emerged as the strongest predictor (OR 1.87 per month), consistent with existing literature (26–28). A higher platelet count predicted chronicity (OR 1.36). This seemingly paradoxical finding may reflect that patients presenting with moderate thrombocytopenia (20–50 × 10⁹/L) often have insidious onset and underlying immune dysregulation, whereas those with severe thrombocytopenia (<10 × 10^9^/L) frequently experience acute, infection-triggered ITP with higher spontaneous remission rates (29). A history of infection within one month (IOV) was protective (OR 0.72), suggesting that infection-triggered ITP may resolve following pathogen clearance (30, 31).

### 4.4 Clinical implementation and practical utility

The model achieved high sensitivity (97.4% at the 0.15 threshold) and negative predictive value (94.7%), with modest specificity and limited positive predictive value (PPV 38.5%–64.7% across thresholds, Table 5). These performance characteristics suggest utility primarily for excluding chronic progression rather than confirming it.

A two-tier risk framework may facilitate interpretation of model outputs in clinical settings, distinguishing a low-risk tier (nomogram score 0–20, predicted probability <15%) from an elevated-risk tier (score >20, probability ≥0.15). Several applications for this tool in clinical practice can be envisioned.

First, risk stratification could potentially help minimize the frequency of laboratory monitoring in low-risk patients relative to higher-risk counterparts, given the high negative predictive value (94.7%) and sensitivity (97.4%) at the 0.15 threshold. This low-risk tier is expected to capture approximately 40%–50% of patients.

Second, the model may help contextualize decisions regarding the early introduction of second-line therapies. Patients with elevated predicted probabilities might be identified as candidates for closer observation or earlier consideration of alternative treatments, whereas the low-risk group is less likely to require therapeutic escalation. This distinction could also inform clinical trial design; for instance, studies investigating early combination strategies (e.g., thrombopoietin receptor agonists or immunosuppressants with corticosteroids) might prioritize enrollment of higher-risk subgroups identified by this nomogram.

Third, at diagnosis, the nomogram may provide quantitative risk estimates to facilitate shared decision-making and family counseling. For example, a predicted probability of 30% at 12 months can offer a data-informed anchor for discussions regarding expected disease course, independent of immediate therapeutic decisions. Given that approximately 70% of pediatric ITP cases resolve spontaneously, identifying a low-risk subgroup in whom expectant management carries a low probability of chronic progression could help avoid unnecessary treatment with corticosteroids or IVIG, thereby potentially reducing avoidable morbidity and associated healthcare utilization.

### 4.5 Limitations

Given that flow cytometry requires specialized equipment, a simplified four-variable model was developed to improve accessibility. Among the five antibodies tested, only GPIIb was utilized in our final model; future investigations should explore antibody combinations (e.g., GPIIb + GPIIIa) and differentiate between IgG and IgM classes. The single-center retrospective design limits the generalizability of our findings, underscoring the need for multicenter prospective validation. Bootstrap validation may overestimate model performance by 0.05–0.10 AUC points. The sample size [*n* = 381, events = 126, events per variable (EPV) = 31.5] constrains extensive subgroup analysis. Additionally, our model relies solely on baseline data; future models incorporating dynamic treatment response data are warranted. The study experienced a case attrition rate of 55.8% (482/863), primarily due to incomplete medical records (28.6%) and insufficient follow-up (26.3%) in a retrospective design. Although Supplementary Table 1 indicated no significant baseline differences in age, sex, or initial platelet counts between included and excluded patients (*P* > 0.05 for all), we acknowledge that loss to follow-up may disproportionally affect patients with milder disease (who may not return for 12-month visits), thereby potentially inflating the estimated risk of chronicity. This limitation underscores the need for prospective multicenter validation with standardized follow-up protocols. ICD-10 code-based screening is susceptible to coding inaccuracies and misclassification; D69.4 in particular captures a spectrum of non-ITP thrombocytopenias. Although we performed manual validation and excluded confirmed alternative diagnoses, residual misclassification cannot be fully ruled out. This represents a limitation inherent to retrospective database studies.

### 4.6 Future directions

While our logistic regression model offers transparency and clinical interpretability, machine learning approaches may improve predictive performance by capturing non-linear interactions between variables (20).

Such methods typically necessitate larger datasets (*n* > 1,000) to mitigate overfitting, as well as external validation across multiple centers to ensure generalizability. The interpretability of conventional regression models remains advantageous for clinical risk scores.

Prospective multicenter validation is required before clinical implementation. Subsequent studies might also evaluate whether quantitative anti-GPIIb antibody titers improve discrimination, and whether dynamic treatment response data further enhance predictive accuracy.

## 5 Conclusions

We developed a predictive model for chronic ITP in children incorporating the anti-GPIIb antibody, selected via LASSO regression, and validated using Bootstrap methodology. To our knowledge, this study represents the first integration of platelet-specific antibodies into prognostic modeling for pediatric immune thrombocytopenia, providing a practical tool for early risk stratification. Prospective multicenter validation and quantitative antibody detection methods merit further investigation.

## Data Availability

The raw data supporting the conclusions of this article will be made available by the authors, without undue reservation.

## Supplementary material

Supplementary Figure 1 Patient flow diagram.
