# Supplementary figures and images for: Development and validation of a predictive model for chronic or persistent immune thrombocytopenia in children incorporating anti-glycoprotein IIb antibody: a retrospective cohort study utilizing LASSO regression and bootstrap stability analysis
Source: Front Pediatr. 2026 Jun 5;14:1832712. doi: 10.3389/fped.2026.1832712 (PMC13279312; doi:10.3389/fped.2026.1832712)

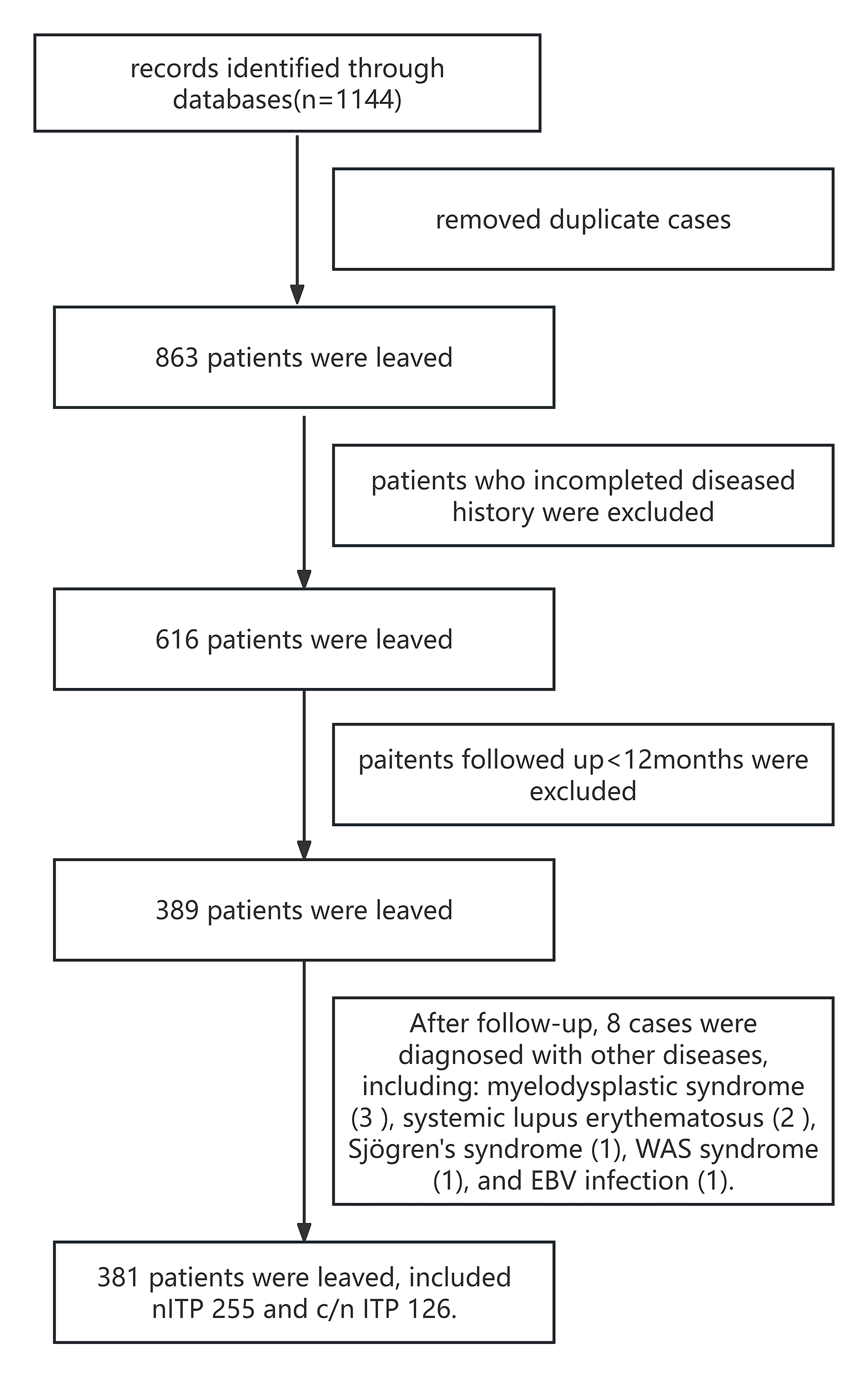

Supplement: Supplementary Figure 1 — Patient flow diagram. [file Image1.tif]
