# Supplementary material for: Development and validation of a predictive model for chronic or persistent immune thrombocytopenia in children incorporating anti-glycoprotein IIb antibody: a retrospective cohort study utilizing LASSO regression and bootstrap stability analysis
Source: Front Pediatr. 2026 Jun 5;14:1832712. doi: 10.3389/fped.2026.1832712 (PMC13279312; doi:10.3389/fped.2026.1832712)
Supplement: Supplementary file 2 [file Table1.pdf]

Supplementary Table 1. Comparison of Basic Patient Parameters Between Exclusion and Inclusion Groups

Variables	excluded(N=474)	included (n=381)	Z/U value	*P* value
sex, n (%)			0.062	0.804
male	285(60.13)	230 (60.37)		
female	189(39.87)	151 (39.63)		
Age, month	35(9.92, 81.00)	36.00 (10.67, 72.00)	-0.024	0.981
PLT, $\times 10^9$/L	12(4.00, 33.00)	11.00 (3.00, 25.00)	-0.650	0.516
ALC, $\times 10^9$/L	3.51(2.46, 5.97)	3.78 (2.56, 5.92)	-0.235	0.814
