# Supplementary material for: Development and validation of a predictive model for chronic or persistent immune thrombocytopenia in children incorporating anti-glycoprotein IIb antibody: a retrospective cohort study utilizing LASSO regression and bootstrap stability analysis
Source: Front Pediatr. 2026 Jun 5;14:1832712. doi: 10.3389/fped.2026.1832712 (PMC13279312; doi:10.3389/fped.2026.1832712)
Supplement: Supplementary file 3 [file Table2.pdf]

Supplementary Table 2. Sensitivity analysis and evaluation of core variables

variable	MICE_Bayesian	MICE_Point	Simple_Median	Simple_Mean	$\bar{x}$±SD	CV(%)	Robustness rating
age	97.4	97.2	97.8	97.6	97.5±0.3	0.3	High stability
IOV	97.2	96.8	95.4	96.2	96.4±0.8	0.8	High stability
GPIIb	95.9	94.2	93.8	94.8	94.7±0.9	0.9	High stability
PLT	95.9	93.6	92.4	92.0	93.5±1.7	1.8	High stability
TT	90.5	90.8	91.2	90.6	90.8±0.3	0.3	High stability
C4	88.3	85.4	82.6	83.2	84.9±2.6	3.0	High stability
sex	89.1	87.6	86.4	87.6	87.7±1.1	1.3	High stability
GMP140	85.8	84.2	82.4	81.8	83.6±1.8	2.1	High stability
HPAb	**87.6	62.4	62.2	62.2	68.6±12.7	**18.5	Less stable
APPT	80.5	76.8	74.2	67.0	74.6±5.6	7.5	Stability
ALC	73.5	68.4	66.2	65.8	68.5±3.6	5.2	Stability
IgG	64.6	58.2	54.4	49.2	56.6±6.6	11.7	Less stable

Footnote: IOV, history of infection or vaccination within 4 weeks preceding ITP diagnosis; GPIIb, glycoprotein IIb; PLT, platelet count; TT, thrombin time; C4, complement component 4; GMP140, P-selectin; HPAb, *Helicobacter pylori* antibody; APTT, activated partial thromboplastin time; ALC, absolute lymphocyte count; IgG, immunoglobulin G. CV=coefficient of variation, calculated as SD/Mean × 100%. High stability: CV<5%; Stability: CV 5-10%; Less stable: CV>10%.
