# Supplementary material for: Development and validation of a predictive model for chronic or persistent immune thrombocytopenia in children incorporating anti-glycoprotein IIb antibody: a retrospective cohort study utilizing LASSO regression and bootstrap stability analysis
Source: Front Pediatr. 2026 Jun 5;14:1832712. doi: 10.3389/fped.2026.1832712 (PMC13279312; doi:10.3389/fped.2026.1832712)
Supplement: Supplementary file 4 [file Table3.pdf]

Supplementary Table 3: Performance comparison of candidate prediction models with varying stability-based variable combinations

Model	N_vars	EPV	Apparent _AUC	Optimism _AUC	Corrected _AUC	95% CI	AIC	Corrected _Brier
**Stability_Top 3**	3	29.3	0.674	0.021	0.654	（0.515，0.796）	327.28	0.217
**Stability_Top 4**	4	22.0	0.695	0.031	0.664	（0.527，0.801）	321.89	0.214
**Stability_Top 5**	5	17.6	0.714	0.040	0.674	（0.539，0.808）	314.38	0.210
**Stability_Top 6**	6	14.7	0.727	0.048	0.679	（0.544，0.814）	311.64	0.210
**Stability_Top 7**	7	12.6	0.745	0.054	0.692	（0.555，0.828）	306.77	0.207
**Stability_Top 8**	8	11.0	0.765	0.063	0.702	（0.569，0.835）	302.35	0.207
**Stability_Top 9**	9	9.8	0.771	0.096	0.702	（0.570，0.835）	302.04	0.208
**Stability_Top 10**	10	8.8	0.775	0.075	0.700	（0.567，0.833）	303.46	0.210
**Stability_Top 11**	11	8.0	0.776	.0.83	0.693	（0.562，0.824）	304.08	0.213
**Stability_Top 12**	12	7.3	0.776	0.091	0.684	（0.555，0.813）	305.37	0.217
**Stability_Top 13**	13	6.8	0.777	0.099	0.678	（0.548，0.809）	307.04	0.220
**Stability_Top 14**	14	6.3	0.779	0.107	0.673	（0.543，0.802）	308.78	0.224
