# Supplementary material for: Development and validation of a predictive model for chronic or persistent immune thrombocytopenia in children incorporating anti-glycoprotein IIb antibody: a retrospective cohort study utilizing LASSO regression and bootstrap stability analysis
Source: Front Pediatr. 2026 Jun 5;14:1832712. doi: 10.3389/fped.2026.1832712 (PMC13279312; doi:10.3389/fped.2026.1832712)
Supplement: Supplementary file 5 [file Datasheet1.pdf]

Supplementary Methods 1

Analytical Pipeline for Model Development and Validation

S1.1 Overview of the Multi-Phase Analytical Framework

The model development and validation pipeline comprised four sequential phases, implemented exclusively in the training set for variable selection and internal validation, with the independent test set reserved solely for final performance evaluation.

Phase 1: Univariate screening ($P < 0.10$) → LASSO regression with 10-fold cross-validation.

Phase 2: Bootstrap stability assessment ($B = 1{,}000$, λ.1se criterion) to identify highly stable predictors (selection frequency ≥ 90%).

Phase 3: Candidate model comparison using bootstrap optimism correction and the Equivalent Parsimony Principle (ΔCorrected AUC < 0.01).

Phase 4: Final independent evaluation on the test set ($n = 115$).

All analyses were conducted in Python 3.9 using scikit-learn (v1.3.0), statsmodels (v0.14.0), and pROC (v1.18.0). Random seed = 42 was fixed for all stochastic procedures to ensure reproducibility.

S1.2 Multiple Imputation by Chained Equations (MICE)

S1.2.1 Imputation Strategy

Missing data in the training set were imputed using MICE with $m = 5$ imputed datasets and maxit = 50 iterations per dataset. The imputation model specification was:

• Continuous variables: Predictive Mean Matching (PMM, $k = 5$ nearest neighbors) to preserve distributional properties and avoid implausible values.

• Binary/categorical variables: Logistic regression (binary) or multinomial logistic regression (categorical with >2 levels).

Convergence was assessed by examining trace plots of mean and standard deviation across iterations for key variables (PLT, ALC, age). No systematic trends were observed after iteration 20, indicating adequate convergence.

S1.2.2 Test Set Imputation

To maintain strict separation between training and test data, missing values in the test set were imputed using predictive means derived from the training set's imputation model. Specifically, the imputation equations estimated from each of the 5 training imputed datasets were applied to the test set, yielding 5 test-set imputations. Final test-set predictions were averaged across these 5 versions.

S1.2.3 Sensitivity Analysis of Imputation Methods

Four imputation strategies were compared to assess robustness:

1. MICE_Bayesian: Bayesian linear regression as the imputation engine for continuous variables.
2. MICE_Point: Standard MICE with predictive mean matching (as used in the primary analysis).
3. Simple_Median: Missing values replaced by the median (continuous) or mode (categorical) of the training set.
4. Simple_Mean: Missing values replaced by the mean of the training set.

The coefficient of variation (CV = SD/mean) for core prognostic factor regression coefficients was calculated across methods. CV < 5% for age, PLT, IOV, and GPIIb confirmed robustness. HPAb showed higher sensitivity (CV = 18.5%), leading to its exclusion from the final model.

S1.2.4 Pooling Rules

Results from the 5 imputed training datasets were combined using Rubin's rules:

• Point estimates: Arithmetic mean of the 5 dataset-specific estimates.

• Variance: Within-imputation variance + Between-imputation variance + Between-imputation variance/m (total variance = $\bar{V} + B + B/m$).

S1.3 LASSO Regression and Variable Selection

S1.3.1 Standardization

All continuous predictors were standardized to zero mean and unit variance (Z-score normalization, standardize = TRUE) before LASSO fitting to ensure penalty equivalence across variables with different scales.

S1.3.2 Cross-Validation Setup

LASSO logistic regression was fitted with 10-fold cross-validation:

• Stratification: Folds were stratified by outcome (c/pITP vs. nITP) to maintain outcome prevalence balance in each fold.

• Lambda selection: Two criteria were evaluated: – λ.min: The lambda value yielding the minimum mean cross-validated deviance. – λ.1se: The largest lambda value within 1 standard error of the minimum deviance (the primary criterion used in this study for parsimony).

• Elastic net mixing parameter: $\alpha = 1.0$ (pure LASSO, no ridge component).

S1.3.3 Variable Inclusion Rule

Variables with non-zero coefficients at λ.1se were retained for subsequent bootstrap stability assessment. Variables compressed to exactly zero were considered excluded by the penalty.

S1.4 Bootstrap Stability Assessment

S1.4.1 Resampling Protocol

A non-parametric bootstrap procedure with $B = 1{,}000$ resamples was performed on the training set ($n = 266$):

• Each bootstrap sample was generated by sampling with replacement (n = 266 per sample).

• MICE was reapplied to each bootstrap sample (m = 5, maxit = 50) to account for missing data uncertainty within resampling.

• LASSO logistic regression (λ.1se, 10-fold CV) was fitted on each imputed bootstrap sample.

S1.4.2 Stability Frequency Calculation

For each candidate predictor, the selection frequency was defined as the proportion of bootstrap resamples (out of 1,000) in which the variable received a non-zero coefficient at λ.1se.

Stability classification:

• Highly stable: Selection frequency ≥ 90%.

• Moderately stable: Selection frequency 80–89%.

• Unstable: Selection frequency < 80%.

Only highly stable predictors were considered for candidate model construction.

S1.4.3 Bootstrap OR and Coefficient Distribution

The mean and standard deviation of regression coefficients across the 1,000 resamples were calculated for each highly stable variable. Bootstrap odds ratios (OR) were derived by exponentiating the mean coefficients. The 95% bootstrap confidence intervals were estimated using the percentile method (2.5th and 97.5th percentiles of the bootstrap distribution).

S1.5 Bootstrap Optimism Correction and Candidate Model Comparison

S1.5.1 Optimism Correction Procedure

For each candidate model, bootstrap optimism correction was performed to obtain internally validated (corrected) performance estimates:

1. Fit the model on the original training set → calculate Apparent AUC (optimistic estimate).

2. For each of B = 1,000 bootstrap samples: a. Fit the model on the bootstrap sample. b. Calculate AUC on the bootstrap sample (bootstrap performance). c. Calculate AUC on the original training set (test performance). d. Optimism = Bootstrap AUC − Test AUC.

3. Mean optimism = Average optimism across 1,000 resamples.

4. Corrected AUC = Apparent AUC − Mean optimism.

S1.5.2 Candidate Model Definition

Eight candidate models were compared, incorporating combinations of highly stable predictors and clinically relevant variables:

Table S1. Candidate prediction models and variable composition

**Model**	**Variables**	**Rationale**
TOP8	Age + IOV + PLT + GPIIb + C4 + TT + sex + IgA	All highly stable predictors
TOP9	TOP8 + GP140	Adds GMP140
TOP10	TOP8 + GP140 + PLR	Adds GMP140 and PLR
**M-4A**	Age + IOV + PLT + GPIIb	**Final model**: parsimonious + clinically feasible
M-4B	Age + IOV + PLT + sex	Replaces GPIIb with sex for settings without antibody testing
M-5A	Age + IOV + PLT + GPIIb + TT	Adds TT (high stability, less specific)
M-5B	Age + IOV + PLT + GPIIb + sex	Adds sex (marginal significance)
M-6	Age + IOV + PLT + GPIIb + TT + sex	Full 6-variable model
M-8	Age + IOV + PLT + GPIIb + TT + sex + C4 + GP140	Expanded model

S1.5.3 Equivalent Parsimony Principle

The Equivalent Parsimony Principle was defined as selecting the model with the fewest predictors among those with ΔCorrected AUC < 0.01 relative to the highest-performing model. This balances predictive performance with model simplicity to minimize overfitting and enhance clinical usability.

S1.5.4 DeLong Test Implementation

Pairwise AUC comparisons between M-4A and alternative models were performed using the DeLong test on the training set predicted probabilities. The test statistic was calculated using the covariance matrix of the rank-correlated AUC estimates (DeLong et al., 1988). A two-sided $P < 0.05$ was considered statistically significant.

S1.6 Threshold Selection and Diagnostic Metrics

S1.6.1 Optimal Threshold

The Youden index (J = Sensitivity + Specificity − 1) was used to identify the optimal probability threshold in the test set. The threshold maximizing J was defined as the operating point for primary reporting.

S1.6.2 Clinical Threshold Exploration

Additional thresholds were explored to accommodate varying clinical decision contexts:

• High-sensitivity threshold for ruling out chronicity (screen-out strategy).

• Optimal threshold by Youden index.

• High-specificity threshold .

S1.6.3 Diagnostic Metrics Calculation

For each threshold, the following metrics were calculated in the test set:

• Sensitivity, Specificity, PPV, NPV, Accuracy

• Likelihood ratios: LR+ = Sensitivity / (1 − Specificity); LR− = (1 − Sensitivity) / Specificity

• Net benefit (DCA) = True Positives / N − False Positives / N × (P_t / (1 − P_t))

S1.7 Nomogram Construction

The final logistic regression coefficients were linearly transformed to a 0–100 point scale:

• Points = ($\beta_i \times X_i - \min(\beta_i \times X_i)$) / ($\max(\beta_i \times X_i) - \min(\beta_i \times X_i)$) × $Point\_range_i$

• Total score = Σ $Points_i$

• Predicted probability = $1 / (1 + \exp(-(\beta_0 + \Sigma\beta_i X_i)))$

Risk tiers were defined by total score cutoffs corresponding to predicted probabilities:

• Low risk

• Moderate risk

• High risk
